# A Move in the Right Direction

**Published:** 1868-01

**Authors:** 


*A Move in the Right Direction.*—We have before us the announcement of a course of special medical instruction, by Dr. H. R. Storer and other physicians of Boston. We find the subjects treated of to consist of Public Hygeine and Medical Jurisprudence, Diseases of Women, Operative Ophthalmology, Diseases of the Skin, Veterinary Surgery and Medicine, Diseases of the Teeth, Surgical Deformities, Diseases of the Throat, Diseases of Infants and Children, Toxicology, Venereal Diseases and Practical Pharmacy. This is a goodly array of specialities. While we might desire to see some omitted, and others inserted in the catalogue, it is nevertheless evident that the student can derive great benefit from the course, if it be faithfully followed. Of course, no one student is likely to take all the tickets. The very minuteness of the detail would prevent that,

So rapid has been the advance of science of late years, that no one can hope to cultivate with success the whole wide field which lies before him. He must be content with the general results of even one study, and confine himself to some special department of it, if he would be thorough, and especially if he would rise to eminence. In nothing is this concentration more necessary than in medicine and surgery. So close and minute has been the study of the numerous acute observers whose attention has been drawn to the healing art, that almost every organ has a literature of its own, which requires no little time and attention to master. This conviction is forcing itself on both the profession and the public. The consequence is that specialists are becoming daily more and more numerous, as well as more necessary.

We therefore gladly chronicle the movement already inaugurated in Boston, and trust it may be followed by others all over the country. Why cannot such a school be organized in Baltimore? We have between three and four hundred medical and dental students here, a respectable number of whom would doubtless be glad to pursue special studies.

